# Discovery and investigation of natural Diels–Alderases

**DOI:** 10.1007/s11418-021-01502-4

**Published:** 2021-03-08

**Authors:** Kenji Watanabe

**Affiliations:** Department of Pharmaceutical Sciences, University of Shizuoka, Shizuoka, 422-8526 Japan

**Keywords:** Diels–Alder reaction, Biosynthesis, Polyketide synthase, Nonribosomal peptide synthetase, Reaction mechanism

## Abstract

It has been proposed that biosyntheses of many natural products involve pericyclic reactions, including Diels–Alder (DA) reaction. However, only a small set of enzymes have been proposed to catalyze pericyclic reactions. Most surprisingly, there has been no formal identification of natural enzymes that can be defined to catalyze DA reactions (DAases), despite the wide application of the reaction in chemical syntheses of complex organic compounds. However, recent studies began to accumulate a growing body of evidence that supports the notion that enzymes that formally catalyze DA reactions, in fact exist. In this review, I will begin by describing a short history behind the discovery and characterization of macrophomate synthase, one of the earliest enzymes that was proposed to catalyze an intermolecular DA reaction during the biosynthesis of a substituted benzoic acid in a phytopathogenic fungus *Macrophoma commelinae*. Then, I will discuss representative enzymes that have been chemically authenticated to catalyze DA reactions, with emphasis on more recent discoveries of DAases involved mainly in fungal secondary metabolite biosynthesis except for one example from a marine streptomycete. The current success in identification of a series of DAases and enzymes that catalyze other pericyclic reactions owes to the combined efforts from both the experimental and theoretical approaches in discovering natural products. Such efforts typically involve identifying the chemical features derived from cycloaddition reactions, isolating the biosynthetic genes that encode enzymes that generate such chemical features and deciphering the reaction mechanisms for the enzyme-catalyzed pericyclic reactions.

## Introduction

**Fig. 1:**
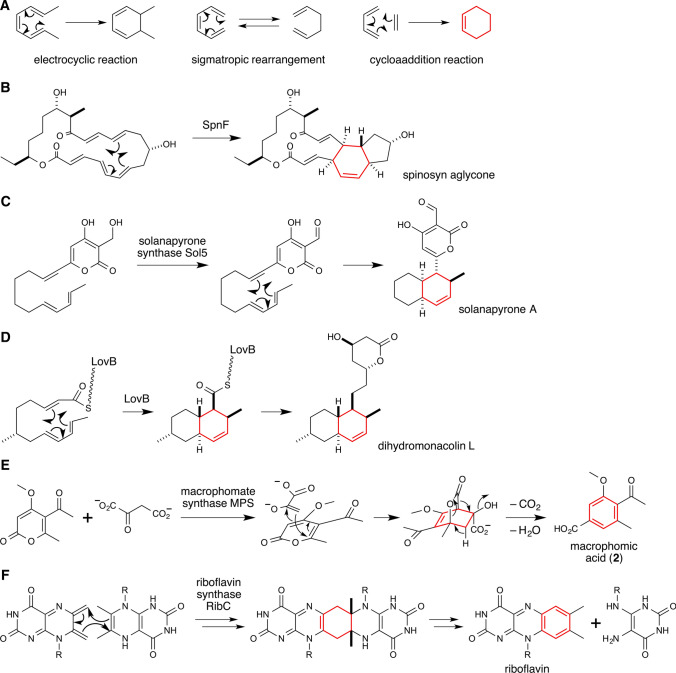
Reaction schemes of biosynthetic transformations proposed to involve a Diels–Alder reaction and the enzyme indicated to catalyze the reaction. **a** General schematics of electrocyclic reaction, sigmatropic rearrangement and cycloaddition reaction, the three main classes of pericyclic reactions. **b** SpnF for spinosyn A biosynthesis. **c** Solanapyrone synthase Sol5 for solanapyrone A biosynthesis. **d** LovB for production of dihydromonacolin L during lovastatin biosynthesis. **e** Macrophomate synthase MPS for macrophomic acid biosynthesis. **f** Riboflavin synthase RibC for riboflavin biosynthesis. The carbon frameworks considered to be generated by Diels–Alder reactions are highlighted in red

To date, much effort has been invested into studying how natural products are biosynthesized, and vast achievements have been made to understanding how these compounds acquire their structural complexity and biological activities. In recent years, significant progress has been realized due to devoted efforts from scientists in this field and rapid advancement in the development of new and powerful technologies. Numerous insightful and innovative discoveries regarding biosynthesis of secondary metabolites have been made by scientists using genetic, molecular biological, biochemical and biophysical techniques. In this review, I present a brief overview and recent update on the study of a class of highly pursued biosynthetic proteins called Diels–Alderases (DAases) that catalyze Diels–Alder (DA) reactions. DA reaction is a type of organic reaction that is categorized as a subset of pericyclic reactions. Pericyclic reactions are reactions that proceed via a single transition state with cyclic geometry, where bonds are formed and broken in a concerted manner [1]. The chemical transformation is often associated with significant structural changes. This class of reactions can be divided into three large categories (Fig. 1a): (1) electrocyclic reaction, which converts a conjugate double bond in a straight chain compound into a cyclic compound with a π-electron system, or a reversed ring opening reaction, (2) sigmatropic rearrangement, which creates one σ-bond by dismantling another σ-bond while simultaneously moving a substituent from one part of the π-bond system to another by means of an intramolecular reaction, and (3) cycloaddition reaction, which affords a single cyclic adduct from overlapping π-orbitals of two unsaturated moieties to provide two σ-bonds in the resulting adduct at the expense of two π-bonds. DA reaction is a specific subset of cycloaddition reaction. It is often referred to as a [4+2] cycloaddition reaction, because a conjugated diene with four π-electrons and a dienophile containing a double or triple bond with two π-electrons combine to form a substituted cyclohexene adduct. Apart from those three classes of reactions, pericyclic reactions also encompass ene reactions as well as the corresponding “retro” or microscopic reverse reactions [2, 3]. Advancement of the theoretical understanding of the pericyclic processes was achieved through the application of the principle of orbital symmetry conservation that led to the development of the Woodward–Hoffmann rules [1, 4–7]. Pericyclic reactions are often analyzed with correlation diagrams that track the evolution of the molecular orbitals of the reacting species that overlap in a continuous cycle through the transition state based on their symmetry properties. Another approach for analyzing the transition state of a pericyclic reaction is based on the frontier molecular orbital theory [8], where the interactions of the highest occupied molecular orbital (HOMO) and the lowest unoccupied molecular orbital (LUMO) are considered.

Pericyclic reactions are very useful in organic chemistry, where a wide range of synthetic applications have been developed [9–12]. Nevertheless, identification and characterization of native enzymes that catalyze pericyclic reactions, especially DA reactions, in living organisms have proven to be challenging. Until recently, only a relatively limited number of examples of natural enzymes that catalyze pericyclic reactions have been described in detail through mechanistic studies. The representative example is chorismate mutase that transforms prephenate into chorismate via a [3,3]-sigmatropic Claisen rearrangement [13–15]. However, identification of additional enzymes that are considered to catalyze DA reactions began to be reported more recently. Discovering novel enzymes and deciphering their catalytic mechanisms in general can be an arduous undertaking that requires an integration of knowledge and technologies cultivated in the fields of biochemistry, natural product chemistry, molecular biology, genetics and even structural biology. Regardless, such enzymes can shed light on how biology accomplishes complex chemical synthesis, providing potential hints for innovation in our drug discovery efforts. In particular, identification of enzymes capable of catalyzing DA reactions would be of particular interest, both from the perspectives of biosynthetic studies and industrial applications. It has been proposed that natural product biosynthesis likely employs DA reactions much more frequently than currently recognized [16–19]. Notwithstanding, so far only five examples of biotransformation and its reaction mechanism have been examined in details using purified natural enzymes that catalyze a DA reaction based on their observed ability to promote a cycloaddition reaction. They are SpnF in spinosyn biosynthesis (Fig. 1b) [20], solanapyrone synthase Sol5 (Fig. 1c) [21], LovB from lovastatin biosynthesis (Fig. 1d) [22, 23], macrophomate synthase MPS for the biosynthesis of macrophomic acid, a substituted benzoic acid (Fig. 1e) [24, 25], and riboflavin synthase RibC involved in riboflavin biosynthesis (Fig. 1f) [26, 27]. SpnF has been described as the first monofunctional enzyme for which a specific acceleration of a [4+2] cycloaddition reaction was verified experimentally as the sole observable activity of the enzyme (Fig. 1b) [20]. One of the key mechanistic aspects of a DA reaction is that the cyclization reaction shall proceed in a concerted manner [28]. To this end, detailed mechanistic studies are still on-going to establish whether or not the cycloaddition reaction catalyzed by SpnF proceeds concertedly [29–31]. LovB, a fungal highly reducing polyketide synthase (PKS), has been shown in vitro to catalyze the formation of the specific *endo* diastereomeric cycloaddition adduct having the identical stereochemistry as dihydromonacolin l that is different from nonenzymatically formed *endo* or *exo* adducts (Fig. 1d) [22]. There have been recent discoveries of a series of enzymes catalyzing the formation of similar polycyclic carbon frameworks, which will be discussed in depth below. Macrophomate synthase MPS has been studied extensively by this author and his colleagues as the first DA-catalyzing enzyme (Fig. 1e) [25, 32] and will be discussed in detail below. Other multifunctional enzymes Sol5 (Fig. 1c) and RibC (Fig. 1f) have been proposed to catalyze [4+2] cycloaddition reactions in addition to a hydroxyl oxidation [21] and a hydride transfer [27], respectively. In this review, I will discuss a handful of enzymes that have been characterized and chemically authenticated to catalyze DA reactions. I will describe the history of discovery and characterizations of MPS further in the first section. Then, I will focus on more recent discoveries of DAases involved in fungal secondary metabolite biosyntheses. Through the discussion, I hope to illustrate that the combination of theory and experiments has played a central role in the recent discovery of new natural products containing features derived from cycloaddition reactions and identification of biosynthetic mechanisms involving enzymes catalyzing pericyclic reactions.

## Macrophomate synthase, a natural Diels–Alder reaction-catalyzing enzyme

Reports from the early 1980’s and subsequent isotope-labeling experiments described the biotransformation of a conjugated 2-pyrone **1** and another unidentified substrate [33, 34] into a corresponding substituted benzoic acid termed macrophomic acid **2** (Fig. 1e) by a phytopathogenic fungus, *Macrophoma commelinae* [33, 35–37]. Subsequently, screening for the formation of **2** from a mixture of **1** and different candidates for the second substrate using various enzyme preparations, such as cell-free extract of the fungal culture [38] and chromatographically enriched crude sample of enzymes [39], in combination with the use of isotope-labeled precursor-feeding experiments led to identification of oxaloacetic acid as the second substrate and Mg^2+^ as an obligatory co-factor [39]. Once we established the reaction condition for the enzyme, we were able to purify the enzyme [24], which was named macrophomate synthase (MPS) [39]. The purified enzyme provided the terminal amino acid sequences used for cloning the *mps* gene [24]. The gene was overexpressed in *Escherichia coli* to prepare an ample quantity of highly pure protein required for further characterizations of the enzyme by means of in vitro assays.

**Fig. 2:**
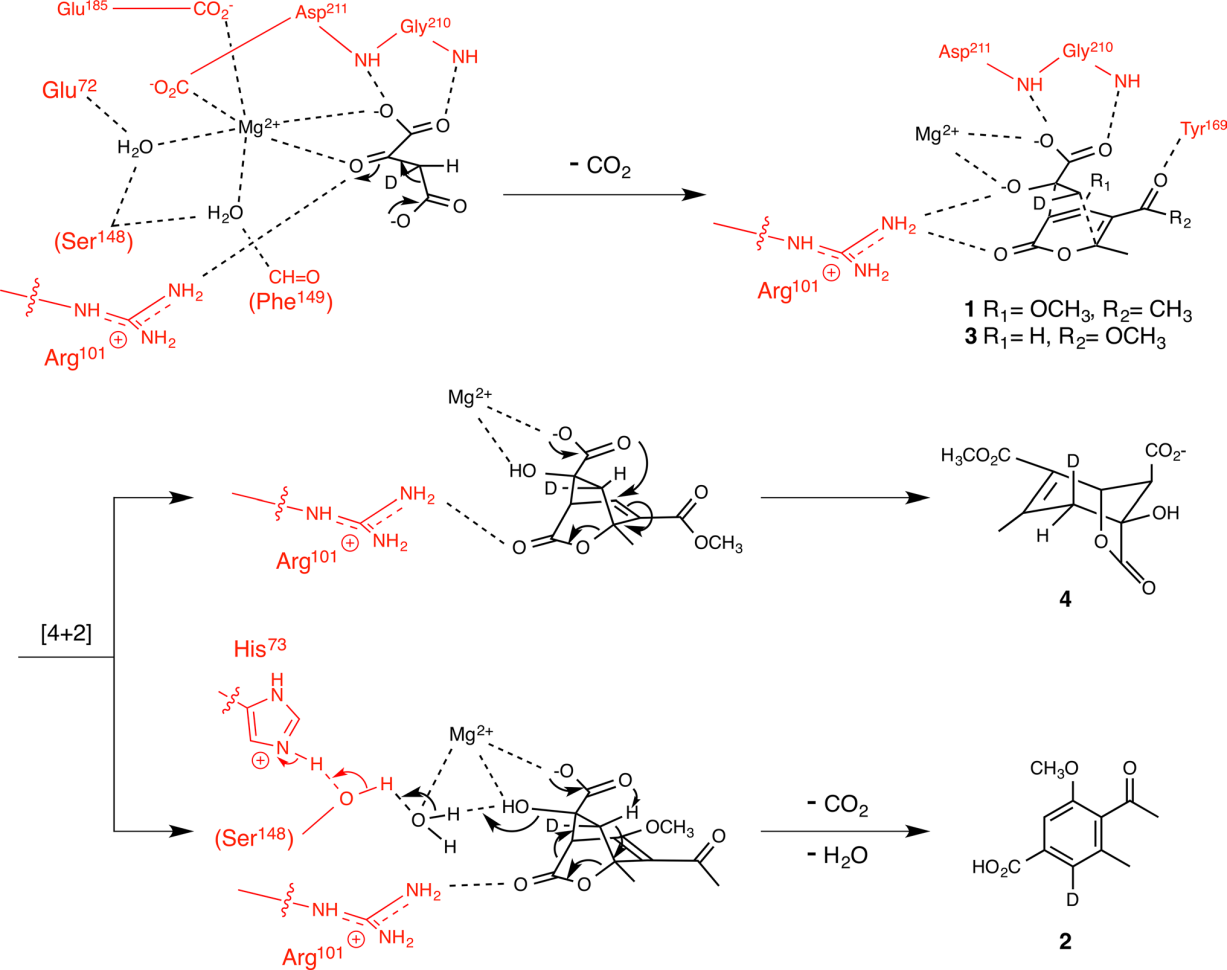
Detailed reaction mechanism proposed for macrophomate synthase showing the interactions between modeled substrates and reaction intermediates and the amino acid residues in the active site and the bound magnesium ion visualized in the crystal structure. The enzyme sidechain groups are in red

While testing the recombinant enzyme for its substrate tolerance using **3** as a 2-pyrone substrate analog, we were not able to obtain the expected corresponding benzoic acid but instead isolated an unexpected bicyclic product **4** from the reaction mixture (Fig. 2) [25, 40, 41]. This surprising finding and determination of the chemical structure of **4** paved the way to conclusively decipher the reaction mechanism this unusual enzyme employs. To understand how **4** is formed and verify it as a shunt product of a DA reaction in the absence of the original substrate **1**, we elucidated the stereochemical course of the complete reaction by determining the absolute configuration of **4** using deuterium-labeled oxaloacetic acid which we prepared using an enzyme coupled reaction [25]. The ability of this enzyme to catalyze a DA reaction was advocated, because a set of bicyclo[2.2.2]-intermediate analogs that we designed and synthesized were observed as its competitive inhibitor [42]. These inhibitors were designed to mimic a proposed intermediate that would form during the enzyme-catalyzed DA reaction. Furthermore, detailed kinetic analyses of the enzyme revealed that oxaloacetate was converted to pyruvate enolate via decarboxylation as the first step of the series of enzymatic transformations (Fig. 2, first step). Then, pyruvate enolate undergoes two carbon–carbon bond formations with the 2-pyrone substrate **1** through a DA reaction as a highly efficient step of this enzymatic reaction to form a bicyclic intermediate similar to **4** (Fig. 2, second step). Lastly, another round of decarboxylation and a dehydration take place to form the final product **2** (Fig. 2, third step) [25]. Through the study, we built a strong case to argue that MPS is the first enzyme known to catalyze a DA reaction. More recently, Hilvert and his co-workers elucidated the existence of a late-stage reaction intermediate. They also determined that the final step involving the dehydration reaction to be a non-enzymatic process [43]. The crystal structure of this protein visually revealed not only the positioning of the substrate **1**, pyruvate and Mg^2+^ within the active site, but also the network of close interactions formed among the substrate molecules, co-factor, and the amino acid residues that line the active site (Fig. 2) [32]. The coordination arrangements between the two substrate molecules deduced from the crystal structure were appropriate for maximizing the overlapping of the molecular orbitals between two substrates captured in the enzyme to promote a DA reaction to proceed. However, the exact nature of the reaction mechanism still remains debated [43, 44], as recent studies have also suggested that tandem Michael–Aldol reaction as another plausible reaction pathway for this transformation [44, 45]. Further studies are required to establish MPS to be a true DAase.

## StmD catalyzes a [6+4] cycloaddition reaction in streptoseomycin biosynthesis

**Fig. 3:**
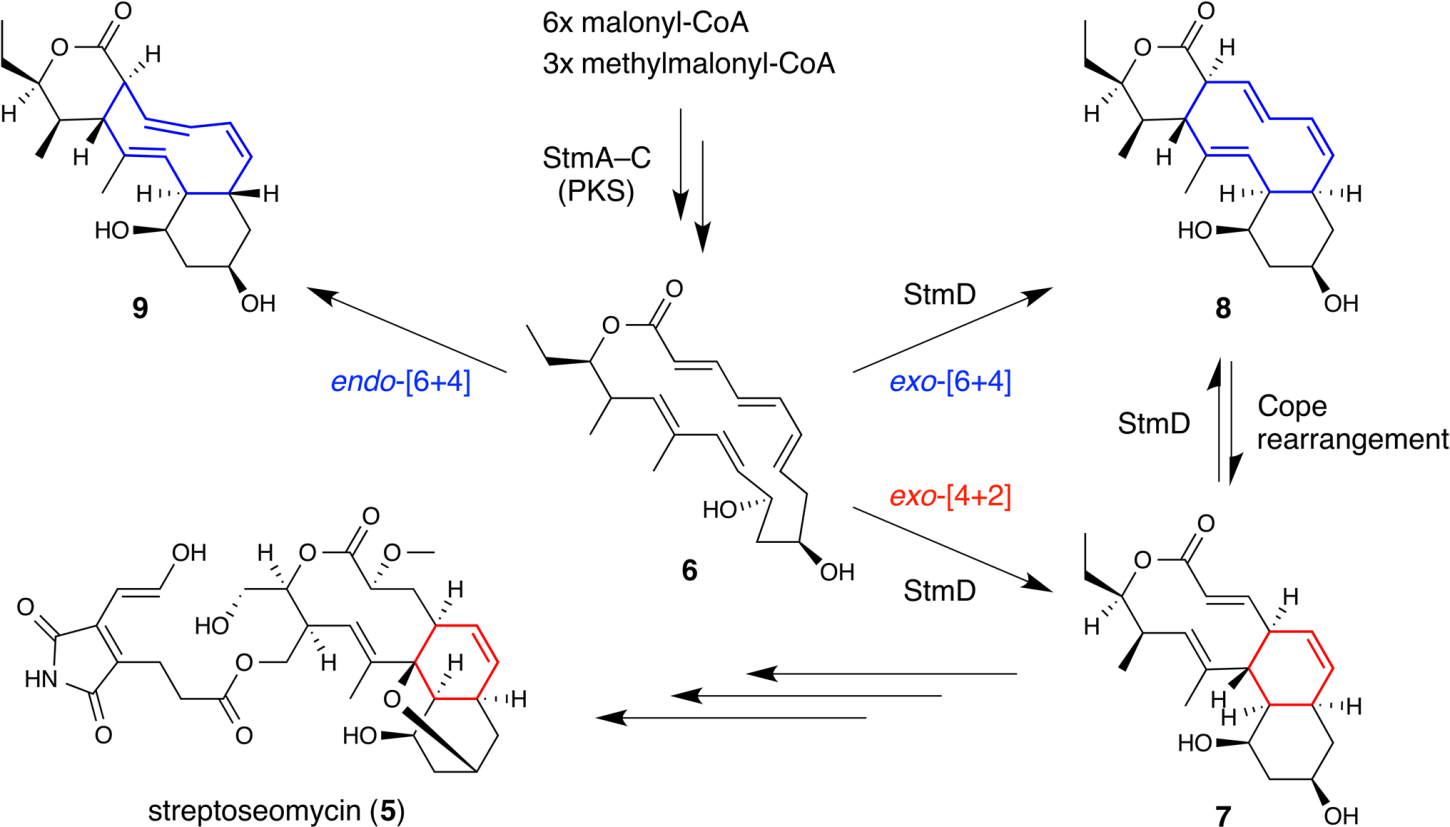
The proposed reaction mechanism for StmD-catalyzed conversion of macrolide polyketide intermediates through an ambimodal cycloaddition reaction in streptoseomycin (**5**) biosynthesis. The carbon bonds considered to be involved in the [4+2] and [6+4] cycloaddition reactions are colored in red and blue, respectively

Streptoseomycin **5** (Fig. 3) produced by a marine streptomycete *Streptomyces seoulensis* is a newly identified macrodilactone natural product with potent activities against microaerophilic bacteria [46] that is a member of the family of oxa-bridged macrolide antibiotics such as nargenicin [47]. It contains a complex pentacyclic 5/14/10/6/6 ring system, where the 18-membered 10/6/6 tricyclic lactone moiety is derived from a macrolide polyketide intermediate **6** that is assembled by a set of type I PKSs StmA–C [46]. Similar to spinosyn A (Fig. 1b) and its proposed biosynthetic mechanism [48], the central cyclohexene unit within the *cis*-decalin system can be readily considered to be the product of a [4+2] cycloaddition reaction catalyzed by a DAase. However, a very recent comprehensive study regarding the characterization of StmD, the pericyclic reaction-catalyzing enzyme of the streptoseomycin biosynthetic pathway, by a combination of extensive experimental and computational analyses indicated that the cyclization of **6** proceeds in a manner more complex than previously proposed during the production of the tricyclic intermediate **7** (Fig. 3) [49].

StmD was identified using a biogenesis-based comparison strategy, an effective approach that has previously been applied in tracing functionally unpredicted enzymes such as monofunctional [4+2] cyclases PyrE3 and PyrI4 in the biosynthetic pathway of pyrroindomycins [50]. StmD shares no sequence homology with SpnF (see the section below on Sch210972 for details), but both operate on a similar ambimodal mechanism, where StmD transforms its substrate **6** into the major *exo*-[6+4] adduct **8** (Fig. 3, blue) along with the minor *exo*-[4+2] adduct **7** simultaneously (Fig. 3, red) [49]. Remarkably, both adducts **7** and **8** can be the intermediates in the biosynthetic pathway of streptoseomycin. Intermediates **7** and **8** interconvert to each other via a facile Cope rearrangement, and the equilibrium tends to lean toward the *exo*-[4+2] adduct side when the downstream biosynthetic enzymes are present. Only a trace level of **9**, the *endo*-[6+4] adduct that is thermodynamically more stable than **7** and **8**, was observed in the StmD-catalyzed cyclization of **6**, indicating the stereoselectivity StmD exerts on the cycloaddition reaction it catalyzes. The observation of the formation of the cycloadducts **8** and **9** is of particular importance, as it provides the experimental evidence of an enzymatic [6+4] cycloaddition reaction for the first time [49]. The crystallization of NgnD, the functionally identical counterpart of StmD from the biosynthetic pathway of the structurally related nargenicin [47], allowed identification of key amino acid residues that were proposed to play a role in the polarization of the transition state that stabilizes the transition state. This mechanistic hypothesis was biochemically validated in vitro through site-specific mutagenesis of the enzyme. Consequently, StmD, along with its homologs such as NgnD, represent a new type of ambimodal pericyclases that are involved specifically in the biosynthesis of the widely distributed streptoseomycin-type of natural products.

## LepI in leporin C biosynthesis catalyzes an “ambimodal reaction” involving both hetero-Diels–Alder and intramolecular Diels–Alder cycloadditions

**Fig. 4:**
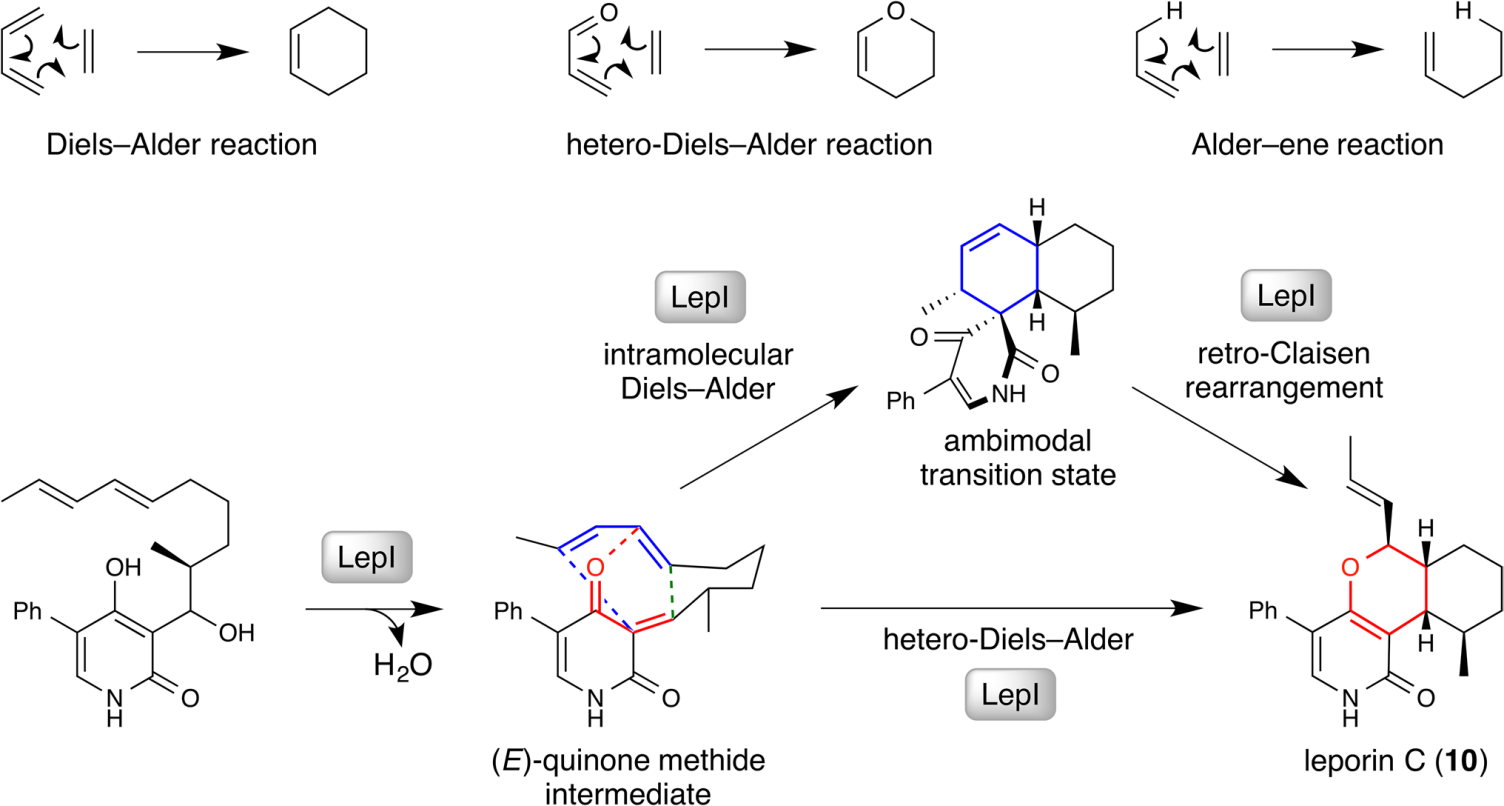
(Top row) Different types of enzymatic pericyclic reactions proposed to be involved in the biosynthesis of fungal natural product, leporin C (**10**). (Bottom row) The proposed biosynthetic steps for the conversion of the quinone methide intermediate into leporin C catalyzed by the Diels–Alderase LepI. Both single-step inverse-electron-demand hetero-Diels–Alder reaction (red) and two-step intramolecular Diels–Alder reaction followed by a retro-Claisen rearrangement (blue) are proposed to be catalyzed naturally by this enzyme

In 2017, Tang et al*.* identified [51, 52] the *S*-adenosylmethionine (SAM)-dependent enzyme LepI, which catalyzes a hetero-Diels–Alder (HDA) reaction (Fig. 4, top row, middle), in a biosynthetic study of leporins produced by the filamentous fungus *Aspergillus flavus* [53]. Based on the experimental results, LepI catalyzes a diastereoselective dehydration reaction of the 2-pyridone alcohol substrate as the first step to form a quinone methide intermediate (Fig. 4, bottom row, first step). Subsequently, LepI transforms the intermediate into tricyclic leporin C **10** by catalyzing not only an HDA reaction but also an intramolecular Diels–Alder reaction (IMDA) followed by a [3,3]-retro-Claisen rearrangement reaction (Fig. 4, bottom row, second and third steps). The transition state of the quinone methide intermediate is considered to form an “ambimodal” transition state in the active site of LepI from which the reaction trajectory can either proceed to the HDA (Fig. 4, bottom row, red) or IMDA (Fig. 4, bottom row, blue) pathway. The multifunctional aspect of this interesting enzyme was analyzed and corroborated with detailed computational studies based on density functional theory calculations and determination of the crystal structures of LepI in complex with ketone analogs of both the substrate and the product **10** [52]. The biosynthetic transformations leading to the formation of **10** adds another example to an emerging small group of biocatalytic steps involved in the biosynthesis of polycyclic natural products such as spinosyn A (Fig. 1b) [54] and heronamide A [55] that are proposed to proceed through ambimodal transition states that can resolve in either a [4+2] or [6+4] cycloadduct formation. Interestingly, SpnF, the enzyme responsible for the cycloaddition reaction involved in the biosynthesis of spinosyn A, is also a SAM-dependent methyltransferase (SAM-MTase) [56]. These results may provide important insights into the evolution of SAM-MTases into multifunctional enzymes that can catalyze ambimodal pericyclic reactions. In fact, identification of such SAM-MTases led to the discovery of a similar enzyme that is capable of catalyzing another type of pericyclic reaction called the Alder-ene (AE) reaction (Fig. 4, top row, right) [57]. The next section will focus this AE-catalyzing enzyme.

## PdxI/EpiI in pyridoxatin/fusaricide biosynthesis: a turning point between Diels–Alder and Alder-ene reaction

**Fig. 5:**
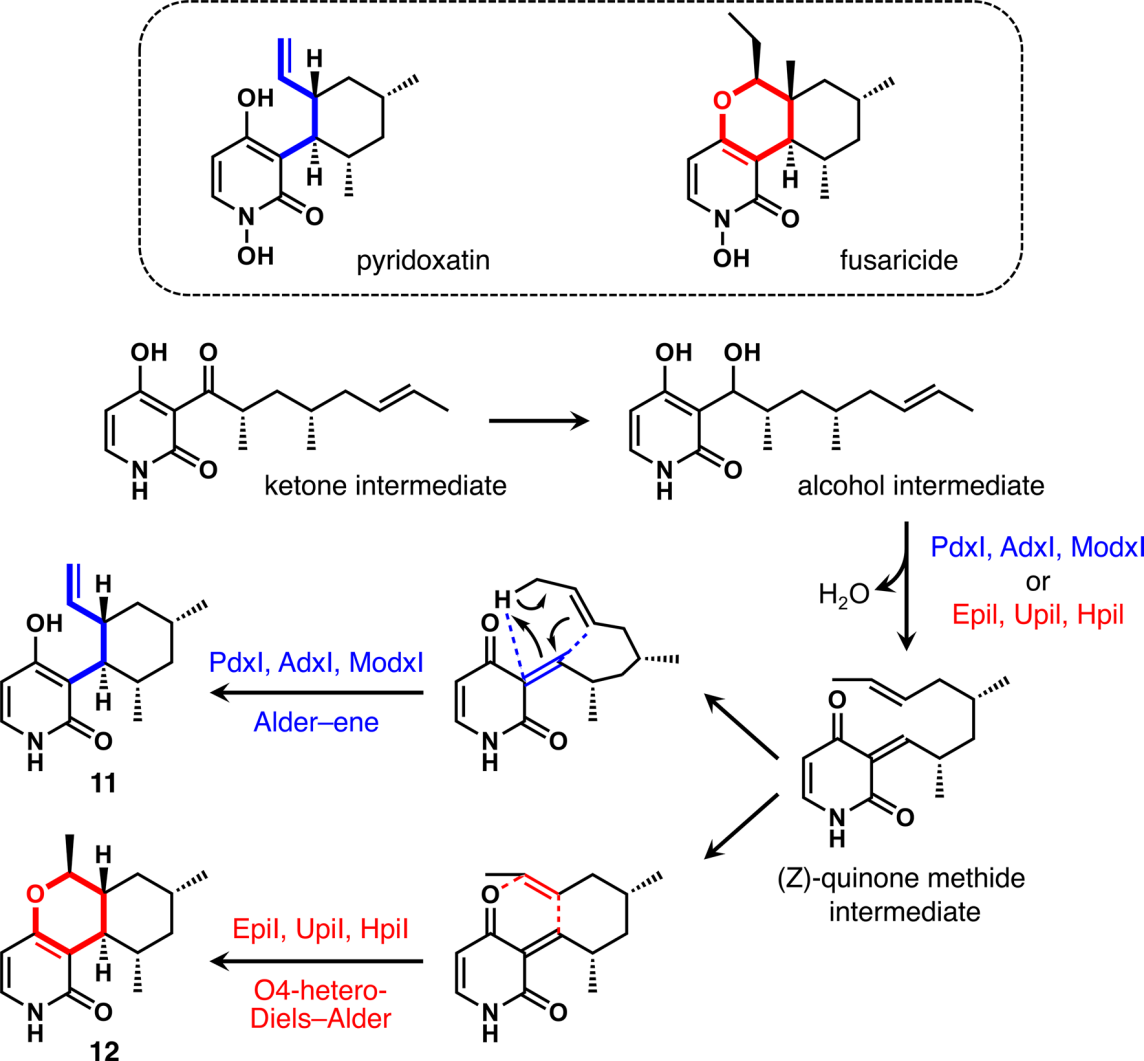
The proposed enzyme-catalyzed formations of the Alder–ene product (**11**) and the hetero-Diels–Alder product (**12**) from the common precursor, (*Z*)-quinone methide intermediate, in the biosyntheses of pyridoxatin/fusaricide. The group of enzymes including PdxI, AdxI and ModxI are considered to catalyze the Alder-ene reaction (blue), while closely related enzymes EpiI, UpiI and HpiI catalyze the hetero-Diels–Alder reaction (red)

Tang et al*.* also focused on the biosynthesis of another group of natural products derived from filamentous fungi called pyridoxatin [58, 59] and fusaricide [60, 61] (Fig. 5, top box). While pyridoxatins and fusaricides are obtained from different species of filamentous fungi, they are considered to be derived from the identical complex substrate [62]. It was proposed that fusaricide was produced by the HDA reaction and pyridoxatin by an AE reaction from the same (*Z*)-quinone methide intermediate (Fig. 5) [57]. Considering the similarities in the core structures of the proposed substrates (the quinone methide intermediates) and the type of reactions being catalyzed (pericyclic reactions), it was hypothesized that the enzymes responsible for the hetero-DA reaction (Fig. 5, red bonds) for fusaricide biosynthesis and the AE reaction (Fig. 5, blue bonds) for pyridoxatin biosynthesis would also belong to the same SAM-MTase family as LepI that catalyzes the [4+2] cycloaddition for the biosynthesis of **10** (Fig. 4, bottom row) [52]. Upon screening the genomes of the pyridoxatin-producing strain *Albophoma yamanashiensis* and the fusaricide-producing strain *Epicoccum sorghinum* FT1062, Tang et al*.* found a SAM-MTases gene in a homologous biosynthetic gene cluster found in each of the genomes and named them *pdxI* and *epiI*, respectively. Although PdxI and EpiI have low amino acid sequence homology with LepI of only approximately 15%, all three proteins belonged to the SAM-dependent *O*-MTase family. When PdxI was reacted with an alcohol intermediate as a substrate, the enzyme promoted an AE reaction following a dehydration reaction to form the pyridoxatin-like product **11** (Fig. 5). On the other hand, EpiI was shown to catalyze an O4-HDA reaction on the same alcohol intermediate as its substrate to generate the fusaricide-like product **12** (Fig. 5). Despite the low amino acid sequence homology of 66% between PdxI and EpiI, it is surprising that the positional selectivity and periselectivity each enzyme imparts on the same substrate are significantly different.

**Fig. 6:**
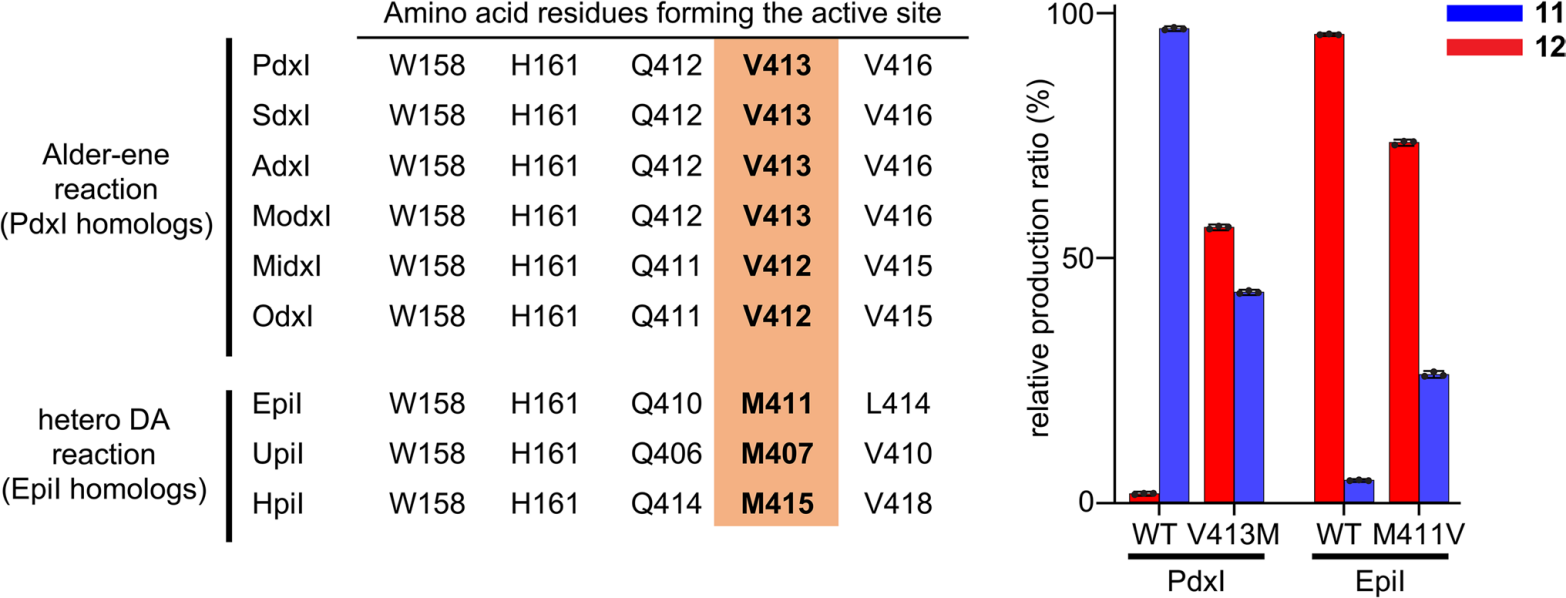
A single active-site residue is the key determinant of the different catalytic modes exhibited by the PdxI vs. EpiI homologs. (Left panel) Amino acid sequence alignment of key active-site residues in PdxI and EpiI homologs. The Alder–ene-catalyzing PdxI homologs and the hetero-Diels–Alder-catalyzing EpiI homologs have almost identical sets of active-site residues except for one position, where the PdxI homologs have a valine residue whereas the EpiI homologs have a methionine residue (highlighted in orange). (Right panel) Exchanging V413 to a methionine in PdxI resulted in greater than 50% loss in the formation of the Alder–ene product **11**, the original product, and a substantial increase in the formation of the hetero-Diels–Alder product **12**. Similarly, replacing M411 to a valine in EpiI led to a decreased formation of **12** and an increased formation of **11**

Tang et al*.* further performed X-ray crystal structure analyses on the co-crystals of PdxI and HpiI (an EpiI homolog) proteins and their corresponding substrate analogs. The structural information revealed that the 17 amino acid residues in the active site of both proteins are identical except for one residue. This amino acid residue was valine in the AE-catalyzing enzymes represented by PdxI (V413), whereas the corresponding amino acid was methionine in the HDA-catalyzing enzymes including EpiI (M411) (Fig. 6, left table). In addition, the lysine residue in the active site of the AE-catalyzing PdxI (K337) was found to form an optimal hydrogen bond (2.8 Å in length) with the C4 oxygen on the pyridone ring of the bound substrate analog, while the geometry of the corresponding hydrogen bond between the active-site lysine residue (K339) in the HDA-catalyzing HpiI and the substrate analog was poor (4.1 Å long). Those key lysine residues are in close proximity to the only active-site residue that varies between PdxI (V413) and EpiI (M411). This valine-to-methionine side chain difference is thought to prevent the optimal hydrogen bond from forming between the lysine residue and the substrate and allow the HDA reaction to proceed. A site-directed mutagenesis study and computational analysis indicated that the ability of this family of enzymes to form the lysine-to-substrate hydrogen-bonding interaction is key to determining the periselectivity, namely the selectivity between HDA and AE reaction, of the enzyme [57]. Conversion of V413 to a methionine residue in the AE-catalyzing PdxI resulted in a reduction of the formation of **11** and a concomitant boost in the formation of **12**. Similarly, mutagenesis of M411 to a valine residue in the HDA-catalyzing EpiI led to a decrease and increase in the production of **12** and **11**, respectively (Fig. 6, right bar graph). Thus, the reported study is a great example of a combination of ligand-complex crystal structure analyses, kinetic characterizations of the wild-type and site-specific mutants, as well as computational studies of reaction mechanisms nicely elucidating how periselectivity and regioselectivity are achieved in those MTase-type pericyclases. Through this and other studies [52, 54, 57], we are starting to gain a better understanding of the molecular basis of substrate recognition and the mechanism of regioselective cyclization in pericyclases including DAases.

There is no clear commonality among the structures of the domains that are responsible for catalyzing DA-type reactions reported to date [32, 49, 52, 56, 57, 63]. The most commonly found folds for DAases thus far are the SAM-MTase fold described above. SpnF [54], LepI [52], PdxI and HpiI [57] belong to this group. However, there is another group of enzymes having a single-domain lipocalin-type fold that catalyze IMDA reactions during the biosynthesis of tetronate and tetramate-containing natural products. Currently known members are PyrI4 [64], AbyU [65] and Tsn11 [66]. Moreover, further structural diversity was observed, as reports of DAases assuming a beta-barrel fold (MPS [32]), AttH-like fold (CghA [67] discussed in the next section), dimeric α + β barrel fold (StmD, NgnD and 101015D [64]), NTF2-like fold (IdmH [68]), and FAD-binding alpha/beta fold having a mixture of a 2-layer and 3-layer alpha/beta/alpha (aba) sandwich architectures (MaDA [63]) have also been reported. This observed diversity in the structures of pericyclases is thought to reflect the substantial differences in the structures of the substrates and minimal requirements for the enzyme active site to provide site-specific functional groups to catalyze pericyclic reactions. Certain pericyclases, such as the SAM-MTase-type pericyclase LepI [52], are proposed to have acquired their pericyclase activities by flexibly evolving the scaffold of existing enzymes with different activities, such as SAM-dependent methyltransfer activities, to confer desired pericyclase activities. In fact, as the study on PdxI and HpiI has shown, only a single active-site amino acid residue change is all that it took to convert an HDA-catalyzing enzyme into an AE-catalyzing enzyme [57]. Those studies have provided innovative insights into the structural basis of the SAM-MTase-type of pericyclases, and we look forward to further development of pericyclase research following the discovery of the world’s first enzyme that catalyzes an AE reaction.

## Sch 210972 biosynthesis involves a Diels–Alder reaction to form the octalin core catalyzed by an enzyme with a fold new to Diels–Alderases.

**Fig. 7:**
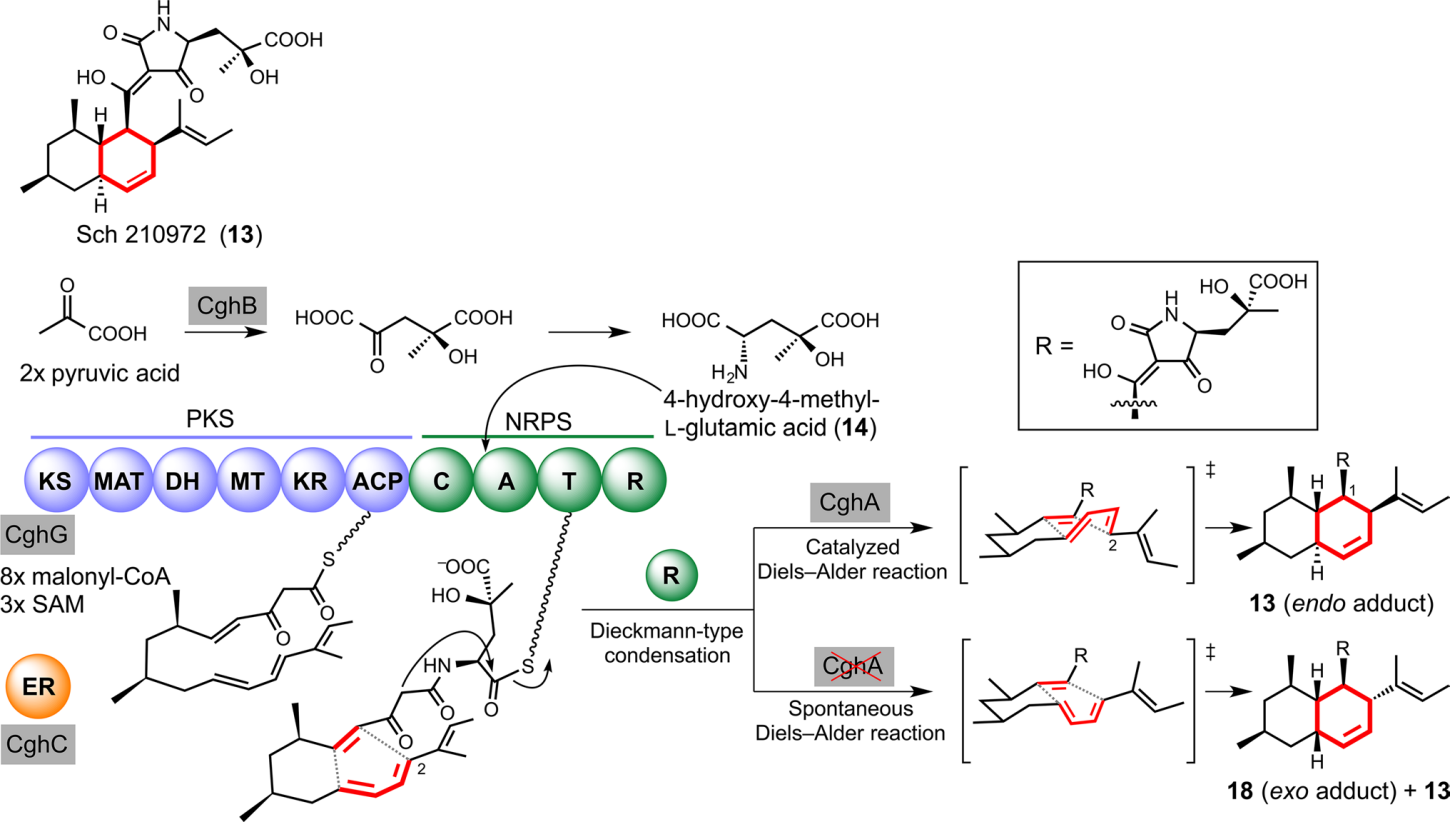
Octalin-containing fungal secondary metabolites and the proposed involvement of Diels–Alder reaction in their biosyntheses, represented by the proposed biosynthesis of Sch210972 (**13**). The hybrid polyketide synthase (PKS)–nonribosomal peptide synthetase (NRPS) CghG and the stand-alone enoyl reductase (ER) CghC together form the linear intermediate. The terminal reductase (R) domain of CghG is proposed to catalyze the tetramate moiety-forming Dieckmann-type condensation. CghA is thought to catalyze a Diels–Alder reaction to form the octalin core, while a non-enzymatic Diels–Alder reaction leads to the formation of the *exo* adduct **18**. The carbon frameworks considered to be involved in the Diels–Alder reactions are highlighted in red. *KS* ketosynthase, *MAT* malonyl-CoA acyltransferase, *DH* dehydratase, *MT* methyltransferase, *KR* ketoreductase, *ACP* acyl carrier protein, *C* condensation, *A* adenylation, *T* thiolation, *SAM* *S*-adenosyl-l-methionine

**Fig. 8:**
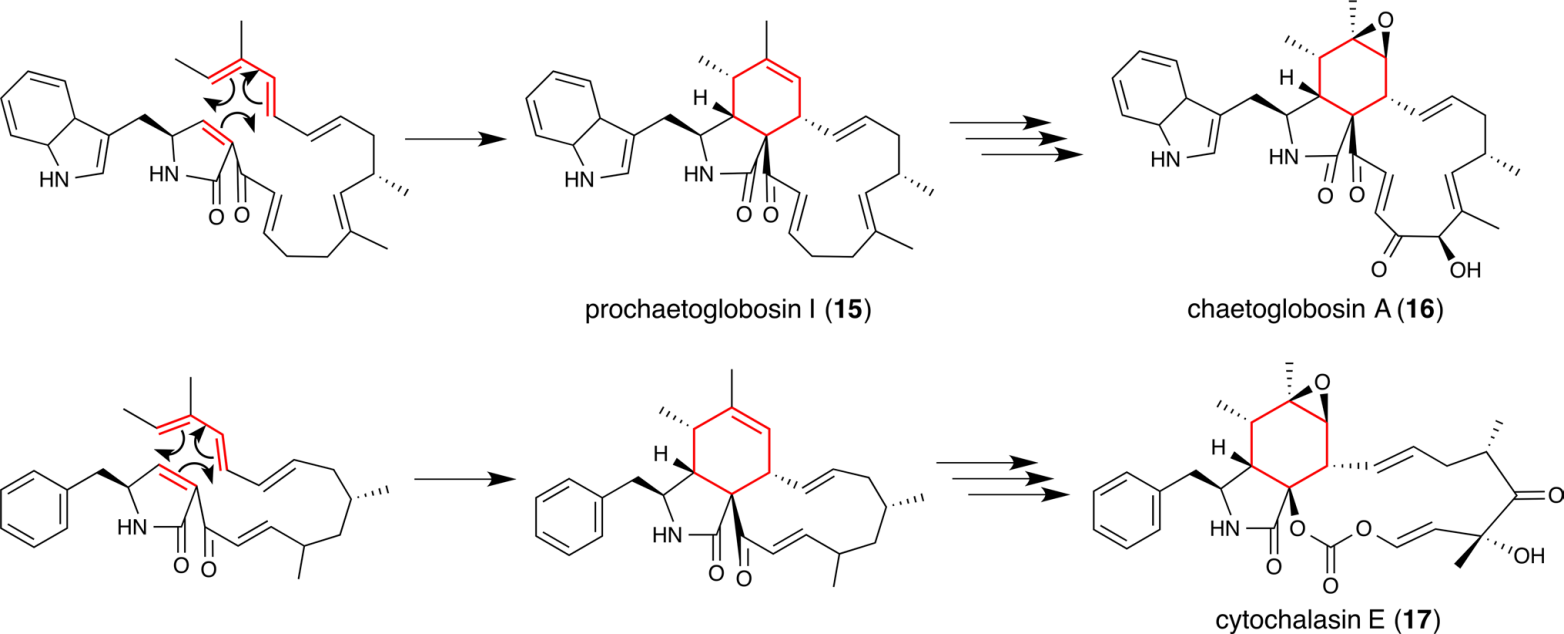
Enzymatic intramolecular Diels–Alder reactions (the first step) predicted to be involved in the biosyntheses of the PK–NRP hybrid precursors leading to the formation of chaetoglobosin A (**16**) and cytochalasin E (**17**)

In our continued search for a *bona fide* DAase, we have focused on a compound called Sch 210972 **13** from *Chaetomium globosum* (Fig. 7, top row). This compound has a unique inhibitory activity against the cell surface receptor CCR-5.16, a biological activity that can be exploited as a novel antiviral treatment against HIV-1 infection by blocking viral cell entry [69]. We were particularly interested in **13**, because it contains an octalin core structure which we speculated to be generated by a DA cycloaddition reaction. We have identified the gene cluster encoding a set of enzymes responsible for the biosynthesis of **13** through genome mining. In addition to an aldolase CghB that is predicted to take part in the formation of 4-hydroxy-4-methyl-l-glutamic acid **14**, an unusual amino acid building block of **13** (Fig. 7, middle row), we confirmed the involvement of a polyketide synthase–nonribosomal peptide synthetase (PKS–NRPS) hybrid megasynthase CghG and a stand-alone enoyl reductase CghC in the formation of the core structure of **13** (Fig. 7, bottom row) through gene disruption in *C. globosum* [70]. This enzymatic arrangement is reminiscent of the involvement of LovB (PKS) and LovC (stand-alone enoyl reductase) for the lovastatin biosynthesis. As described in the introduction, the decalin moiety of the lovastatin core structure has been proposed to be biosynthesized via a DA reaction [22]. Because of the high similarities between the mode of biosynthesis and the chemical structure of the bicyclic C10 core of lovastatin and **13**, we hypothesized that a DA reaction is involved in the biosynthesis of **13**. Similar mechanisms have also been proposed for the PKS–NRPS-catalyzed, DA reaction-mediated formation of the core scaffolding structure of a wide range of compounds, including prochaetoglobosin I **15**, the precursor of chaetoglobosin A **16** (Fig. 8, top row) also from *C. globosum* [71, 72], cytochalasin E **17** from another fungus *Aspergillus clavatus* NRRL 1 (Fig. 8, bottom row) [73], equisetin from *Fusarium heterosporum* [74, 75] and related fusarisetin A from *Fusarium* sp. FN080326 [76], myceliothermophin E from the thermophilic fungus *Myceliophthora thermophile* [77], phomasetin from *Pyrenochaetopsis* sp. RK10-F058 [76], UCS1025A from *Acremonium* sp. KY4917 [78], varicidins from *Penicillium variabile* [79], ilicicolin H from *Cylindrocladium ilicicola* MFC-870 and other fungi [80] and pyrichalasin H from *Magnaporthe grisea* NI980 [81]. Targeted disruption of *cghA* from the Sch 210972 biosynthetic gene cluster in *C. globosum* led to the formation of an *exo* form of the product **18** (Fig. 7, bottom row) as determined by NMR spectroscopy and X-ray crystallography, in addition to the *endo* product **13**, which is the sole product isolated from the wild-type *C. globosum* [70]. These results indicated that CghA controls the diastereoselectivity of the DA reaction that leads to the formation of the octalin core of **13**. Most recently, based on the co-crystal structure of the CghA–**13** complex that we determined at 2.0 Å resolution, a series of site-directed mutants were designed [67]. Biochemical characterizations of the mutants with a simplified substrate analog combined with computational analyses of the enzymatic and non-enzymatic reaction pathways revealed the potential mechanism of how CghA catalyzes the [4 + 2] cycloaddition reaction stereoselectively and avoids product inhibition. Lastly, our rational engineering of CghA based on the structural information allowed conversion of the enzyme to preferentially catalyze the formation of the energetically disfavored *exo* adduct. There are several reports of catalytic antibodies that were designed successfully to catalyze energetically disfavored *exo* DA reactions [82, 83]. However, to the best of our knowledge, this is the first report of a successful engineering of a natural DAase to reverse its stereoselectivity to allow the formation of an energetically disfavored adduct.

Studies from our group and others are extending our knowledge and understanding of the potentially widespread class of natural enzymes that catalyze stereoselective cycloaddition reactions, especially those that are responsible for the biosynthesis of decalin/octalin moieties among natural products. Because of the wide applications of DA reactions in synthetic chemistry, it would be valuable to understand how the enzymes catalyze DA reactions and how we can manipulate the stereoselectivity of DAases, especially toward the formation of energetically disfavored products as demonstrated in our recent report [67]. Such knowledge would help advance our study of natural product biosynthesis, and engineered DAases can be very useful in various bio/synthetic applications. Furthermore, because product inhibition is a common problem in artificially designed DAases [84], the finding from our study could also provide insight into how we can create DAases that can achieve high catalytic turnover.

## Conclusion

In this review, I have briefly summarized a selection of studies conducted over the past 10 years toward identifying and characterizing enzymes with DA activities. The occurrence of enzymes catalyzing DA and other pericyclic reactions in nature has been gradually gaining recognition over the years, indicating that the role of pericyclic reactions in living systems is far from being fully appreciated. One clear conclusion coming out of this review is that there are more natural DAases than we previously thought. As demonstrated through studies of natural product biosynthesis, many strategies used in chemical syntheses of complex natural products where DA reactions serve as a critical step have now been corroborated to have precedence in biosynthesis. There is no doubt that recognition of the existence of natural DAases is largely a result of the recent advent in both the accumulation of knowledge of natural product biosynthesis and technologies for investigating the subject. We now have sufficient knowledge of secondary metabolite biosynthesis to be able to identify and predict with reasonable certainty the precursor and outcome of a formal DA reaction in a biosynthetic pathway, and methods and technologies that allow us to test and verify those hypotheses biochemically, structurally and computationally. Both theoretical and experimental considerations are equally indispensable to the identification of candidate pericyclases. In addition, insights gained from structural investigations of the enzymes in complex with their substrates, products or their analogs, such as specific interactions those enzymes make with their ligands during the catalytic cycle, help advance our mechanistic understanding of how the enzymes catalyze pericyclic reactions. Fast advancements in these aspects of the study explains the current excitement in this field.

In biosynthetic pathways, DA reactions can proceed nonenzymatically with enzymatic assistance initiated by upstream priming reactions and/or driven by downstream transforming reactions toward end products. However, many DA reactions likely depend on enzymes, either monofunctional or multifunctional, or simpler protein templates for stereochemistry control, rate enhancement or both. In the latter cases, methodology development is usually necessary to correlate a biocatalyst with a specific DA reaction, because DAases catalyzing a similar reaction can be highly diverse in origin and evolution as exemplified by DAases involved in decalin and octalin formation. Related enzymes include the multifunctional proteins such as solanopyranone synthase Sol5 [21] (another FAD-dependent enzyme with oxidoreductase and DAase activities, similar to the intermolecular DAase MaDA for chalcomoracin biosynthesis [63]), lovastatin synthase LovB [22] (PKS) and lipocalin-like octalin-forming enzymes, such as CghA [67, 70]. Nature appears to utilize different protein scaffolds to match substrate molecules that share the structural hallmark of a pericyclic reaction but have unrelated overall structures. The co-evolution of proteins and the chemical structures of substrates could be an intrinsic part of expanding biosynthetic pathways for related natural products. As such, the process results in generating many phylogenetically distinct but functionally similar catalysts for pericyclic reactions, which serve as key links among distinct pathways that afford chemically recognizable and pharmaceutically important outcomes.

In addition to playing a central role in the theory and practice of organic chemistry, DA reactions are a subject of basic enzymology in terms of understanding how enzymes are capable of catalyzing diverse reactions under mild conditions at superb rates and with exquisite selectivity through stabilization of reaction transition states. As a part of the investigation, devising artificial design of protein catalysts that can promote DA reactions had long been the focus of the discipline until the recent emergence and recognition of a wide-ranging types of natural DAases. It appears that in many cases both natural and artificial DAases share similar mechanisms for accelerating the reaction rate and controlling the stereochemical outcome of the reaction that employ substrate preorganization that achieves reduction of the entropic cost and/or substrate polarization that leads to the reduction of the activation enthalpy. However, natural enzymes stabilize a pericyclic transition state within their active sites by engaging in highly intricate interactions, whereas current artificial enzymes are often still unable to replicate the level of sophistication to a satisfactory level. Undoubtedly, gaining deeper mechanistical understanding of how nature develops a DAase and incorporates the enzyme into a specific biosynthetic pathway for the biosynthesis of complex natural products would significantly facilitate our efforts toward designing, developing and utilizing artificial DAases. Access to tailored DAases will help us address the synthetic challenges that arise from structural complexity of valuable compounds, and improve the accessibility and efficiency of current organic chemical and synthetic biological schemes, especially in the era of artificial intelligence.
