# Correction to: Discovery and investigation of natural Diels–Alderases

**DOI:** 10.1007/s11418-021-01511-3

**Published:** 2021-04-08

**Authors:** Kenji Watanabe

**Affiliations:** Department of Pharmaceutical Sciences, University of Shizuoka, Shizuoka, 422-8526 Japan

## Correction to: Journal of Natural Medicines 10.1007/s11418-021-01502-4

The article Discovery and investigation of natural Diels–Alderases, written by Kenji Watanabe, was originally published electronically on the publisher’s internet portal on March 8, 2021 without open access. With the author(s)’ decision to opt for Open Choice the copyright of the article changed on March 26, 2021 to © The Author(s) 2021 and the article is forthwith distributed under a Creative Commons Attribution 4.0 International License (https://creativecommons.org/licenses/by/4.0/), which permits use, sharing, adaptation, distribution and reproduction in any medium or format, as long as you give appropriate credit to the original author(s) and the source, provide a link to the Creative Commons license, and indicate if changes were made.

The original article has been updated.

